# Bee venom acupuncture, NSAIDs or combined treatment for chronic neck pain: study protocol for a randomized, assessor-blind trial

**DOI:** 10.1186/1745-6215-15-132

**Published:** 2014-04-21

**Authors:** Byung-Kwan Seo, Jun-Hwan Lee, Pil-Kun Kim, Yong-Hyeon Baek, Dae-Jean Jo, Sanghun Lee

**Affiliations:** 1 Department of Acupuncture & Moxibustion, Kyung Hee University Hospital at Gangdong, #149 Sangil-Dong, Gangdong-Gu, Seoul 134-727, Republic of Korea; 2 Acupuncture, Moxibustion & Meridian Research Group, Medical Research Division, Korea Institute of Oriental Medicine, 1672 Yuseong-daero, Yuseong-gu, Daejeon 305-811, Republic of Korea; 3 Department of Neurosurgery, Kyung Hee University Hospital at Gangdong, #149 Sangil-Dong, Gangdong-Gu, Seoul 134-727, Republic of Korea

**Keywords:** Chronic neck pain, Combined treatment, Bee venom acupuncture, NSAIDs

## Abstract

**Background:**

Chronic neck pain (CNP) is a common painful medical condition with a significant socioeconomic impact. In spite of widespread usage, the effectiveness and safety of combined treatments between conventional and complementary alternative medical treatment modalities has not been fully established in a rigorous randomized clinical trial (RCT). This pilot study will provide the clinical evidence to evaluate the feasibility and refine the protocol for a full-scale RCT on combined treatment of bee venom acupuncture (BVA) and non-steroidal anti-inflammatory drugs (NSAIDs) in patients with CNP.

**Methods/Design:**

This is a randomized, single-blind clinical trial with three parallel arms. Sixty patients between 18 and 65 years of age with non-specific, uncomplicated neck pain lasting for at least three months will be enrolled. Participants will be randomly allocated into the BVA, NSAIDs or combined treatment group. Assessors and statisticians will be blinded to the random allocation. All researchers will receive training to ensure their strict adherence to the study protocol. Patients from the BVA and combined treatment group will be treated with a bee venom increment protocol into predefined acupoints for six sessions over a three week period. BVA intervention is developed through a comprehensive discussion among interdisciplinary spine disorder experts, according to the guidelines of Standards for Reporting Interventions in Clinical Trials of Acupuncture (STRICTA). Patients from the NSAIDs and combined treatment groups will be prescribed loxoprofen (one tablet to be taken orally, three times a day for three weeks). Bothersomeness from CNP measured using a visual analogue scale (VAS) will be the primary outcome assessed at screening, visit two (baseline), four, six, eight (4th week assessment) and nine (8th week assessment) follow-up session. VAS for pain intensity, neck disability index (NDI), quality of life, depressive status and adverse experiences will also be analyzed.

**Discussion:**

Our study results will contribute to feasibility evaluation and to relevant RCT protocol development for a full-scale RCT on combined treatment of BVA and NSAIDs for CNP patients.

**Trial registration:**

This study is registered with the United States (US) National Institutes of Health Clinical Trials Registry: NCT01922466.

## Background

Non-specific chronic neck pain (CNP) is a common debilitating medical condition affecting 30 to 50% of the general population and is most prevalent in middle age [1]. In Korea, the cumulative lifetime prevalence of CNP is 20.8% [2]. CNP causes a significant socioeconomic impact related not only to direct medical costs, but also indirect health care costs and loss of productive capacity [1,3,4]. As a mechanical pain without an accurately identifiable pain source [5], CNP may be related to neck pain, upper extremity pain and headache, but is not related to apparent radiculopathies or myelopathies, including sensory and motor dysfunction [5,6].

Depending on the tailored treatment strategies, various treatment modalities are available for CNP including medication (non-steroidal anti-inflammatory drugs (NSAIDs), serotonin reuptake inhibitors and anti-epileptics), interdisciplinary rehabilitation, physical therapy, education, spinal injection and surgical interventions [7,8]. Although pain-relieving medication is most frequently used to alleviate CNP [9], longterm NSAIDs use is limited due to the risk of side effects and patient intolerance [7].

It is reported that neck pain patients tend to concurrently utilize both conventional treatment and complementary alternative medicine (CAM) in the hopes of more positive effects and safety profile [10]. The use of CAM therapeutic modalities including acupuncture, herbal medicine, herbal acupuncture, cupping and chiropractic manipulation has been increased [11-14]. Bee venom acupuncture (BVA) is one of the most frequently performed pharmacopuncture to alleviate neck pain [15,16]. As an acupoint-stimulating treatment, BVA is utilized for the treatment of neck pain, low back pain, acute ankle sprain, post-stroke shoulder pain, rheumatoid arthritis, lumbar disc herniation and osteoarthritis [16,17]. BVA has been reported to exhibit analgesic, anti-inflammatory, anti-arthritic and anti-cancer effects [18] in experimental animal models [19,20] and in clinical studies [15-17,21,22]. The analgesic effects of BVA are more potent with repetitive administration than with a single treatment [23].

Although combined treatments between conventional and CAM interventions have been frequently utilized, the effectiveness and safety has not been fully investigated in rigorously designed clinical trials. This study may be a preliminary evaluation of the non-inferiority of BVA against NSAIDs, and any synergistic or additional effect of each. The results from this pilot study will provide clinical evidence to evaluate the feasibility for full-scale randomized controlled trial (RCT) on combined treatment of BVA and NSAIDs for CNP.

## Methods/Design

### Trial design

**Figure 1:**
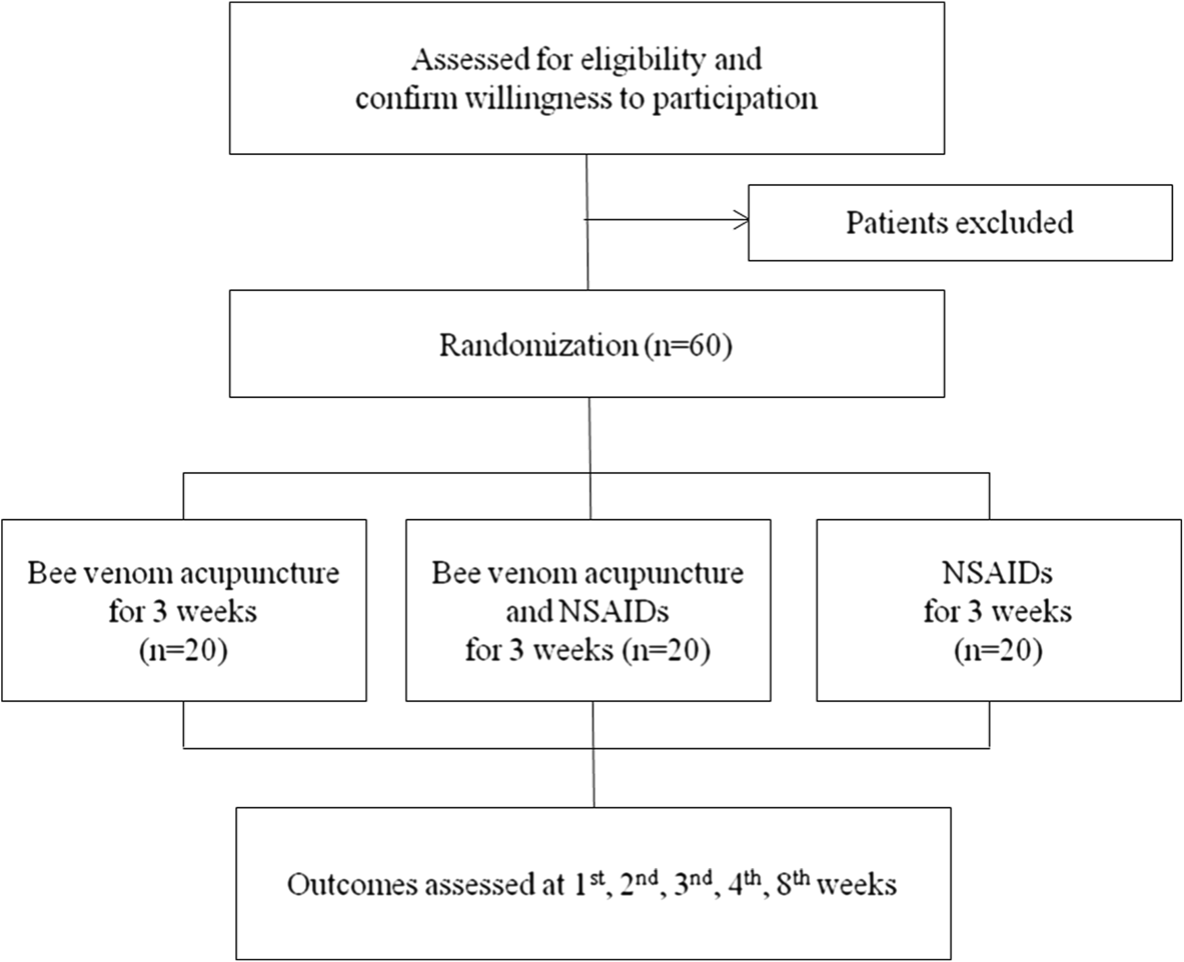
Study flowchart.

This is a randomized, assessor-blind clinical trial with three parallel arms (1:1:1 ratio) conducted at the spine center at Kyung Hee University Hospital at Gangdong (KHUHGD). We will compare the effects of BVA, NSAIDs and the combined treatment of BVA and NSAIDs in patients with CNP. The trial has been registered with the United States (US) National Institutes of Health Clinical Trials registry (NCT01922466) and has been approved by the Institutional Review Board (IRB) of KHUHGD (KHNMC-OH-IRB 2012–019). Outcome assessment and statistical analyses will be performed by independent researchers who are blinded to the patient assignment (Figure 1).

### Participants

Sixty participants with non-specific neck pain will be recruited to the study from KHUHGD through advertisements in local newspapers, on hospital websites and on bulletin boards. All participants will be interviewed by telephone and scheduled for a screening visit by the clinical research coordinator (CRC). After completing the screening questions, participants will be guided through the informed consent process. Participants will be informed that they may be randomly allocated into any of the three treatment groups (BVA, NSAIDs and combined treatment), and that they may withdraw their voluntary participation at any stage. Patients will be provided enough time to deliberate their participation in the independent waiting room with the written information and the informed consent form. As a screening process for eligibility, a skin hypersensitivity test will be performed on each patient at LI11 acupoints, using a subcutaneous injection of 0.05 ml of a 1:20,000 bee venom preparation. Local swelling greater than 10 mm in diameter or redness greater than 20 mm in diameter will be considered a positive reaction, and these patients will be excluded from the study. Eligible participants will be randomly allocated into one of three parallel groups at a ratio of 1:1:1, (BVA:NSAIDs:combined treatment). After random allocation, the interviewer (CRC) will schedule treatment sessions. All recruitment procedures will be recorded in a log file.

### Eligibility criteria

Patients between 18 and 65 years of age who can read and write in Korean, who agree to participate after providing written informed consent, and who have been experiencing non-specific, uncomplicated neck pain for at least three months preceding the time of the screening visit will be selected as study volunteers.

Patients will be excluded from the study if they have: 1) exhibited abnormalities on neurological examination (such as cervical nerve function, deep tendon reflexes, voluntary muscle activation and sensory function); 2) radicular pain; 3) serious spinal disorders including malignancy, vertebral fracture, spinal infection or inflammatory spondylitis; 4) other chronic diseases that could affect or interfere with the therapeutic outcomes including cardiovascular disease, diabetic neuropathy, active hepatitis, fibromyalgia, rheumatoid arthritis, dementia or epilepsy; 5) previous spinal surgery or scheduled procedures during the study; 6) a painful condition induced by a traffic accident; 7) a substantial musculoskeletal problem generating pain from an area other than the neck; 8) conditions for which administration of BVA might not be safe including clotting disorders, administration of an anticoagulant agent, pregnancy and seizure disorders; 9) a documented hypersensitive reaction to previous BVA treatments, bee stings or insect bites; 10) a positive reaction observed during a skin hypersensitivity test; 11) a severe psychiatric or psychological disorder; 12) current use of corticosteroids, narcotics, muscle relaxants or herbal medicines to treat neck pain or any medication considered inappropriate by the investigator; and 13) pending lawsuits or receipt of compensation due to neck pain.

### Interventions

The BVA group will be treated with a bee venom (BV) increment protocol of six sessions over three weeks. The NSAIDs group will be prescribed loxoprofen (loxonin, 60 mg/Tab, Dong Wha Pharm Co., Ltd, Seoul, Korea), at a dose of one tablet administered orally three times per day over three weeks. The combined treatment group will be treated according to the BV increment protocol and will be prescribed loxoprofen over three weeks.

### Bee venom acupuncture

BVA will be prepared as dried bee venom powder (Yoomil Garden, Hwasun, Korea), diluted (1:20,000) and filtered in normal saline (0.9% NaCl) at the Korean medical pharmacy at KHUHGD. BVA treatment will be conducted using 26-gauge sterile disposable syringes (Green Cross Co., Yongin, Korea) containing bee venom diluted with normal saline (0.9% NaCl).

After sterile skin preparation, participants will be administered a perpendicular, subcutaneous injection at a depth of 0.5 to 1.0 cm with the patient lying in the prone position. The acupuncturist will treat participants under the predefined BVA protocol at predefined acupoints. BVA intervention will consist of six sessions twice a week for three weeks, under the following weekly BVA incremental protocol: 0.2 ml for the first week, 0.4 ml for the second week and 0.8 ml for the third week. The predefined points (SI12, SI14, BL11, BL12, TE15 and GB21) have been carefully selected from textbooks and research articles by a process of consensus among participating Korean medical doctors (KMD), all of whom are experienced in the treatment of chronic neck pain.

### Allowance of concurrent treatment of patients

During the study period, all other interventions, drugs and treatments for neck pain will be prohibited including surgical procedures, injections, acupuncture, physical therapy or the use of muscle relaxants, narcotics and analgesics. Regular medications not intended to affect neck-pain related dysfunction will be allowed. Any change in concurrent treatment will be recorded at every visit.

### Outcomes

Assessment of neck pain-related dysfunction, pain and quality of life will be collected five times over eight weeks: at screening, visit two (baseline), four, six, eight (4th week assessment) and nine (8th week assessment) follow-up sessions. Medical history and sociodemographic characteristics, including age, gender, marital status, residence, occupation and education level will be taken from the screening visit. Any unpredicted, adverse events will be recorded at each visit (Table 1).

**Table 1:** Schedule for data collection: outcome measures per visits

**Measures**	**Screening**	**Visit 2 (baseline)**	**Visit 4**	**Visit 6**	**Visit 8 (4th week assessment)**	**Visit 9 (8th week assessment)**
Sociodemographic characteristics	○					
Physical examination	○					○
Laboratory tests	○				○	
VAS for bothersomenesst of CNP	○	○	○	○	○	○
VAS for pain intensity of CNP	○	○	○	○	○	○
Neck disability index		○	○	○	○	○
EuroQol 5-Dimension		○	○	○	○	○
Beck’s depression inventory		○			○	○
SF-36		○			○	○
‘Skin roll’ test		○			○	○
Credibility test		○			○	
Adverse experiences*		○	○	○	○	○

*Adverse experiences will be monitored throughout the clinical trial.

CNP: chronic neck pain; VAS: visual analogue scale.

### Primary outcome measurement

bothersomeness of chronic neck pain will be evaluated using the 10 cm visual analog scale (VAS) for CNP. Participants will be asked to report the degree of clinical severity and impact on activities of daily life experienced within the past week on a continuous discomfort scale of 0 to 10 (0, absence of discomfort; 10, the worst discomfort imaginable) [24] at screening, visit two (baseline), four, six, eight (4th week assessment) and nine (8th week assessment) follow-up sessions. The primary end point will be the VAS score at the four-week follow-up, one week after the completion of the three-week treatment session.

### Secondary outcome measurements

#### VAS for pain intensity

Pain intensity of CNP will be assessed using a 10 cm VAS, a fast and straightforward method for evaluating the subjective degree of pain intensity [25]. Participants will be asked to report the degree of pain intensity using the 10 cm VAS (0, absence of pain; 10, the worst pain imaginable) at screening, visit two (baseline), four, six, eight (4th week assessment) and nine (8th week assessment) follow-up sessions.

#### The neck disability index (NDI)

Neck pain-related dysfunction will be assessed using the validated Korean version of NDI [26] at visit two (baseline), four, six, eight (4th week assessment) and nine (8th week assessment) follow-up sessions. The NDI contains 10 questions about pain intensity, personal care, lifting, reading, headaches, concentration, work, driving, sleeping and recreation. Each question is rated using a 6-point scale from 0 to 5 points (0, absence of problem; 5, disabled by neck pain).

#### The EuroQol 5-dimension

Quality of life (QOL) for patients with CNP will be assessed using the Korean version of the EuroQol 5-dimension (EQ-5D) [27,28] at visit two (baseline), four, six, eight (4th week assessment) and nine (8th week assessment) follow-up sessions. The EQ-5D contains five questions regarding mobility, personal care, daily activities, pain/discomfort and anxiety/depression. Each question is rated on a scale from 1 to 3 points (1, better state of health; 3, worse state of health).

#### Beck’s depression inventory

The depressive status for patients with CNP will be assessed using the Korean version of Beck’s Depression Inventory (BDI) [29,30] at visit two (baseline), eight (4th week assessment) and nine (8th week assessment) follow-up sessions. The BDI contains 21 questions about depressive symptoms rated from 0 to 3 points (the lower the score, the lower the depressive state).

#### SF-36

Health-related QOL for patients with CNP will be assessed using the Korean version of the Medical Outcome Study Short-form 36-Item Health Survey (SF-36) [31,32] at visit two (baseline), eight (4th week assessment) and nine (8th week assessment) follow-up sessions. The SF-36 tests the correlation between health-related QOL and related factors (sex, age, physical function and functioning of daily life) rated on a 5-point scale (1, best status; 5, worst status).

#### Credibility test

The credibility of BVA treatments will be assessed using the credibility test [33] at visit two (baseline) and eight (4th week assessment) follow-up sessions. Participants will rate the credibility of the treatment by answering four questions regarding improvement expected, recommendation to others, rationality of treatment and effectiveness expected for other diseases on a numeric rating scale, with 0 as not at all and 6 as maximal agreement.

#### ‘Skin roll’ test

Painful intervertebral dysfunction will be assessed using the ‘skin roll’ test [34,35] at visit two (baseline), eight (4th week assessment) and nine (8th week assessment) follow-up sessions. The ‘skin roll’ test will be analyzed by an averaging of each of two measured values by two independent assessors: 1) the skin fold thickness measured using a Harpenden skinfold caliper (Baty International, Burgess Hill, United Kingdom); 2) the pain intensity elicited by pressure using a baseline dolorimeter (Fabrication Enterprises. Inc., White Plains, United States); and 3) the subjective pain intensity scored by the VAS.

### Safety

All adverse events and vital signs will be observed and reported according to the standard operating protocol (SOP) of the KHUHGD IRB. Liver and renal functions of each participant will be assessed before the treatment and one week after the end of treatment. A pregnancy test will be performed for female participants of childbearing age at any time required.

### Sample size

Although there are previous researches on the effect of BVA and NSAIDs on chronic neck pain, there are insufficient data to estimate the clinical meaningful minimal change of the combined treatment of BVA and NSAIDs. This study is designed to provide the clinical evidence to estimate the sample size required for a full-scale RCT. As a pilot trial, we will recruit 60 patients into three parallel groups (BVA, NSAIDs or combined treatment). We will assess the impact of the interventions and the safety of combined treatment of different interventions on a limited number of patients.

### Randomization and allocation concealment

A total of 60 participants will be randomly assigned following simple randomization procedures, with a 1:1:1 allocation ratio. The randomization sequence will be generated by an independent researcher using a computerized, random-number generator through a Microsoft Office Excel 2007 software package. Opaque envelopes labeled by study patient number will be conveyed to the interviewer (CRC). The CRC will open the sealed envelopes and allocate participants into the predefined treatment arms. Allocation concealment will not be broken until the completion of the study. The random allocation assignment will be performed only after a participant is confirmed to be eligible and written informed consent has been obtained. Detailed information, including the treatment arm and randomization number from an individual participant will be recorded in the case report form (CRF) and randomization table.

### Blinding

As assessor-blind RCT, the researcher performing the outcome measure assessment will be blinded to each patients’ treatment allocation and will be prohibited from taking part in any conversation about the treatment procedures. It is not possible to conceal BVA practitioners to patient allocations. BVA practitioners will be prohibited from any conversations other than those regarding BVA treatment. NSAIDs will be prescribed by another researcher who will be blinded to the patient allocations and will not be allowed to take part in any conversation regarding the patients' condition. Participants will receive their medication at the KHUHGD pharmacy. Pharmacists will be blinded to the patient allocations and will only be allowed to provide general information regarding medication administration. Statistical analysis will be performed by an independent statistician who will be blinded to patient allocation. All researchers will undergo training prior to participation in this study.

### Education of acupuncturists

Two licensed KMDs who specialize in acupuncture and moxibustion will be designated as the BVA treatment practitioners and will take educational courses to ensure their strict adherence to the study protocol and to become familiar with administering study treatments. In order to ensure consistency in the BVA treatment, the two acupuncturists will undergo intensive and customized training for a full understanding of the BVA increment protocol, including details such as acupuncture points and weekly bee venom dose increments.

### Withdrawal and dropout

Participation in the study will end at any stage if the patient refuses to continue, withdraws consent, violates inclusion or exclusion criteria or the trial protocol, or completes less than four treatment sessions as determined by the attending KMD researchers. The trial will be stopped if the principle investigator believes that there are unacceptable risks of serious adverse events. No interim analysis will be conducted.

### Planned statistical analysis

The analysis will be focused on exploring the feasibility and acceptability of protocol implementation and refining the protocol of future RCT. The results from hypothesis testing will be treated as preliminary and interpreted with caution. The results of this study may provide a preliminary evaluation of any synergistic or additional effect, and of the non-inferiority of combined treatment of BVA and NSAIDs. All statistical analyses will be carried out using the Statistical Package for the Social Sciences (SPSS) for Windows version 18.0 and will be expressed by mean and standard deviation. The level of significance will be set at *P* < 0.05. For each outcome variable, an analysis of covariance (ANCOVA) will be performed on the data to adjust baseline characteristics. Trends over time and time-by-treatment interactions will be explored using a repeated-measures analysis of variance (ANOVA). A Chi-square test or a Fisher’s exact test will be performed to determine differences between groups and adverse effects, which will be recorded and described as a frequency and percentage. Fisher's exact test is used when the cell whose expected value is less than 5 is equal or more than 20%.

### Ethical approval and monitoring

This study has been approved by the KHUHGD IRB (KHNMC-OH-IRB 2012–019; April 2013). Study procedures and documents will be monitored by a qualified clinical research associate (CRA) from the Korea Institute of Oriental Medicine (KIOM).

### Data handling

Data obtained during the study will be confidentially treated according to the standard operation protocol (SOP) of the KHUHGD IRB, and will be stored in lockable cabinets. All data will be processed using the participants’ numbers which will be unrelated to participants’ personal information. Data will be compiled into CRFs and data integration will be thoroughly verified by independent researchers. Non-obvious errors or omissions will be put into data query forms, which will be used for the researchers’ workshop. Data will be gathered and summarized with respect to demographic baseline characteristics, effectiveness and safety observations.

## Discussion

This study will be conducted in a pragmatic setting which is considered appropriate to evaluate a combined treatment of BVA and NSAIDs as it is used in our usual practice [36]. According to the clinical experience, some patients with chronic pain tend to complain of more discomfort and disability than pain intensity itself [37]. Also, a previous study has shown that discomfort can be a more accurate responsive pain measure than pain intensity or other pain measures [38]. Considering the responsiveness study and clinical relevancy, clinical changes will be evaluated using the VAS for discomfort as a primary outcome measure.

The BVA increment protocol and the selected acupoints have been rigorously designed and chosen by a process of consensus among experts according to the Standards for Reporting Interventions in Clinical Trials of Acupuncture (STRICTA) [39]. All study procedures will be performed by delegated researchers trained to meet the study protocol and good clinical practice guidelines. In order to conceal the random allocation in this assessor-blind design, an independent assessor will be appointed and strictly educated to comply with the study protocol. Data will be compiled by an independent researcher, verified through a predefined database quality control procedure and analyzed by an independent statistician.

Although it is designed as a pilot study, every step of clinical study implementation will be rigorously conducted, monitored and supervised to ensure methodological integrity and scientific validity. The results from this pilot study will provide clinical evidence to evaluate feasibility and to develop a relevant RCT protocol on the combined treatment of BVA and NSAIDs for CNP patients. This study is designed to determine whether combined treatment of BVA and NSAIDs is beneficial shortly after the end of the treatment, whether a cumulative effect can be expected from BVA repetition, whether any possible clinical impact will persist, whether patients can take any synergistic advantages, and whether patients will be satisfied with this clinical setting. The findings in this study can be used to calculate effect size and analyze confounding factors for full-scale RCTs on the combined treatment of BVA and NSAIDs for CNP.

### Trial status

This study began in July 2013 after IRB approval. Trial completion is expected by July 2014.

## Abbreviations

ANCOVA: analysis of covariance; ANOVA: analysis of variance; BDI: Beck’s depression inventory; BVA: bee venom acupuncture; CAM: complementary and alternative medicine; CNP: chronic neck pain; CRA: clinical research associate; CRC: clinical research coordinator; CRF: case report form; EQ-5D: EuroQol 5-dimension; IRB: institutional review board; KHUHGD: Kyung Hee university hospital at Gangdong; KMD: Korean medical doctors; NDI: neck disability index; NSAIDs: non-steroidal anti-inflammatory drug; QOL: quality of life; RCT: randomized clinical trial; SF-36: medical outcome study short-form 36-item health survey; SOP: standard operating protocol; SPSS: statistical package for the social sciences; STRICTA: Standards for Reporting Interventions in Clinical Trial of Acupuncture; VAS: visual analogue scale.

## Competing interests

The authors declare that they have no competing interests.

## Authors’ contributions

BKS is responsible for developing the treatment protocol, carrying out and supervising the clinical study and drafting the manuscript as a primary investigator. DJJ and JHL contribute to the study conception and design and participate in the acquisition of data and the revision of the manuscript. PKK contributes to the study implementation and the revision of the manuscript. YHB and SL contribute to the design of the study and supervise the protocol fulfillment and the acquisition of funding for the study. All authors have read, revised and approved the final manuscript.
